# In vitro modeling of renal injury-induced cardiac effects using human iPSC-derived organoids

**DOI:** 10.1186/s12964-026-02902-3

**Published:** 2026-05-06

**Authors:** Beatrice Gabbin, James Gallant, Fangchen Liu, Hailiang Mei, Berend J. van Meer, Ton J. Rabelink, Christine L. Mummery, Jessica M. Vanslambrouck, Cathelijne W. van den Berg, Viviana Meraviglia, Milena Bellin

**Affiliations:** 1 Department of Anatomy and Embryology, Leiden University Medical Center, Leiden, The Netherlands; 2 The Novo Nordisk Foundation Center for Stem Cell Medicine (reNEW), Leiden University Medical Center, Leiden, The Netherlands; 3 Einthoven Laboratory of Vascular and Regenerative Medicine, Leiden University Medical Center, Leiden, The Netherlands; 4 Department of Internal Medicine-Nephrology, Leiden University Medical Center, Leiden, The Netherlands; 5 Department of Biomedical Data Sciences, Leiden University Medical Center, Leiden, The Netherlands; 6 The Novo Nordisk Foundation Centre for Stem Cell Medicine (reNEW), Murdoch Children’s Research Institute, Melbourne, Australia; 7 Department of Paediatrics, Faculty of Medicine, Dentistry and Health Sciences, The University of Melbourne, Melbourne, Australia; 8 Department of Biology, University of Padua, Padua, Italy

**Keywords:** Kidney organoids, Cardiac microtissues, Cardiorenal axis, Inter-organ interaction, Drug-induced toxicity

## Abstract

The bidirectional communication between the heart and kidney is essential for physiological homeostasis, with injury in one organ often impairing the other. Although cardiorenal crosstalk is clinically relevant in conditions such as cardiorenal syndrome (CRS), the underlying molecular and cellular mechanisms remain poorly understood, and in vitro models are lacking. Here, we developed a co-culture system using human induced pluripotent stem cell (hiPSC)-derived kidney organoids (kOs) and cardiac microtissues (cMTs) to model the cardiorenal axis.

kOs exposed to nephrotoxic compounds for 72 h displayed glomerular and tubular damage, reduced cell viability, and altered gene expression. When subsequently co-cultured with cMTs for 72 h, injured kOs induced secondary cardiac dysfunction characterized by reduced cell viability, impaired contractility, and endothelial cell loss. These findings demonstrate that kidney injury can elicit detrimental effects on cardiac tissues in vitro. This organoid-based platform offers a valuable tool for studying cardiorenal interactions and underlines the potential of multi-organ models for investigating mechanisms of interdependent organ dysfunction.

**Supplementary Information:**

The online version contains supplementary material available at 10.1186/s12964-026-02902-3.

## Introduction

The interplay between the heart and kidney, referred to as the cardiorenal axis, is a tightly regulated, bidirectional relationship that plays a central role in maintaining systemic body homeostasis [1]. Dysfunction in either organ can initiate a cascade of maladaptive responses, that ultimately compromise the function of both systems. This interdependence is clinically evident in syndromes such as cardiorenal syndrome (CRS), where in CRS type 3, acute kidney injury (AKI) leads to sudden cardiac injury, while in CRS type 4, chronic kidney disease (CKD) contributes to progressive heart failure [1, 2]. AKI may result from ischemic, septic or nephrotoxic insults, including drugs such as aminoglycoside antibiotics and chemotherapeutics, while CKD typically arises from longstanding conditions such as diabetes, hypertension or glomerulonephritis [3, 4]. In mouse models, doxorubicin has been shown to induce oxidative stress and apoptosis in renal glomeruli and proximal tubules [5, 6], whereas gentamicin triggers tubular necrosis, inflammation and interstitial fibrosis [7]. While the clinical manifestations of heart-kidney crosstalk are well-documented, the molecular and cellular mechanisms underlying these interactions are only partially understood.

Human induced pluripotent stem cells (hiPSCs) have enabled the development of organoid models that recapitulate tissue-specific features of various organs, including the heart [8] and kidney [9]. These organoids offer platforms to model human development, physiology and disease, particularly when integrated into microphysiological systems [10–12]. Dual-organ platforms and organ-on-chip systems are emerging as a new approach to study interorgan communication in vitro under controlled conditions [13]. Our previous study demonstrated the utility of a dual-organ chip to assess heart-kidney crosstalk [14]. However, this work described system integration and hemodynamic simulation rather than a detailed interrogation of the reciprocal effects of injury in one organ on the other.

Here, we build upon these foundational approaches by combining hiPSC-derived kidney organoids (kOs) and cardiac microtissues (cMTs) in a co-culture system that models the human cardiorenal axis. After establishing the independent structural and functional maturation of both organoid types, we induced kidney injury using nephrotoxic compounds and assessed the downstream impact on co-cultured cardiac tissue. Our data shows that damaged kOs negatively affect cMTs, demonstrating that interorgan crosstalk can be captured in vitro using organoid systems and highlight the potential of multi-organ models for studying human disease mechanisms.

Our work aligns with a growing body of literature on capturing interorgan communication in health and disease using hiPSCs either as assembloids or in linked co-cultures, including studies on the liver-heart [15, 16], gut-kidney [17], and brain-heart axes [18]. By providing a mechanistic link between kidney injury and cardiac dysfunction in a human cell model, our study offers new insights into the pathophysiological dynamics of the cardiorenal axis and sets the stage for the development of targeted interventions.

## Results

### Nephrotoxic drugs lead to cell death, tissue remodeling and reduced albumin uptake in kidney organoids

**Fig. 1:**
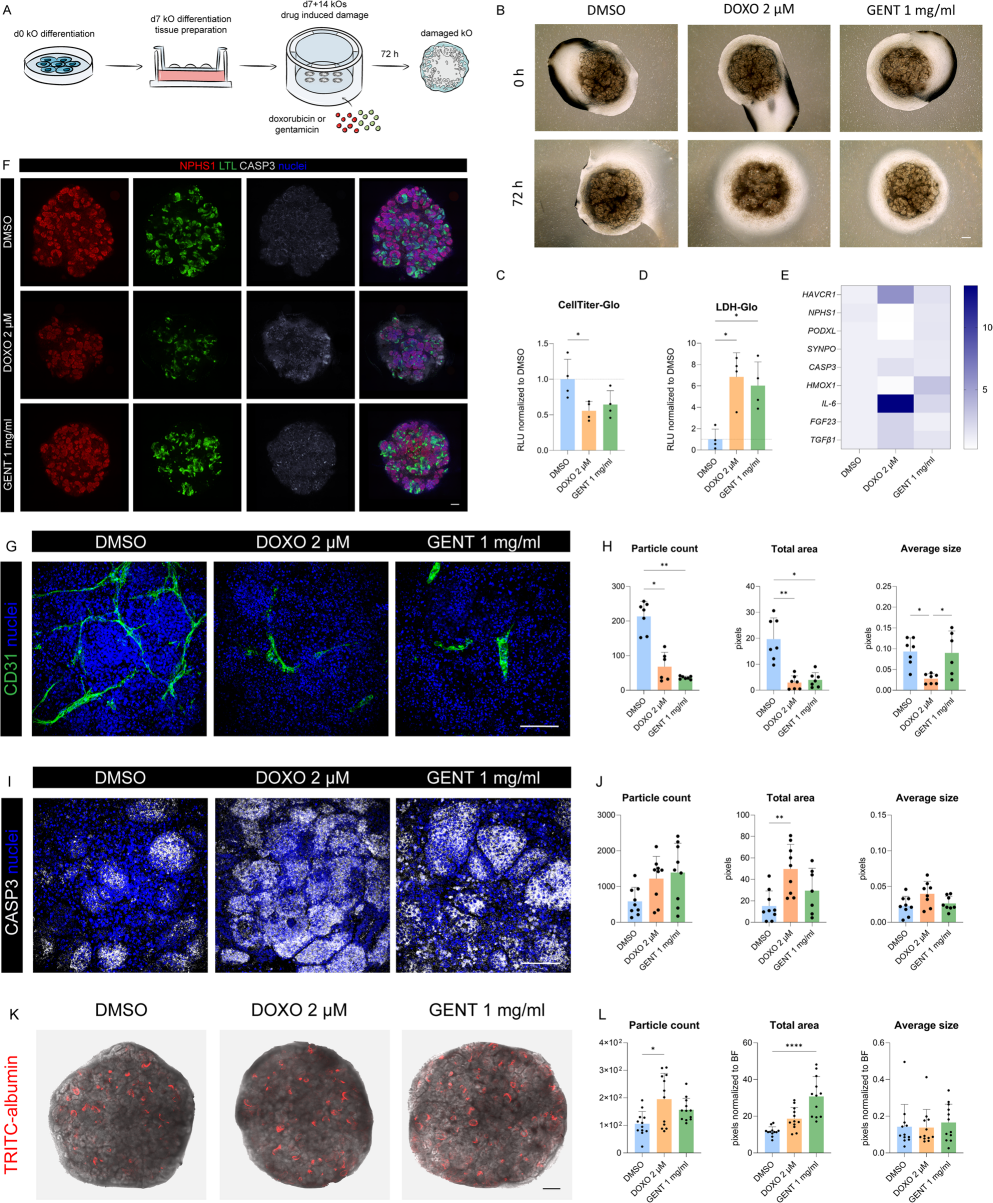
Nephrotoxic drugs lead to reduced cell viability, inflammation and reduced albumin uptake in kOs. **A** Schematic representation of the experimental workflow to induce kO damage using nephrotoxic drugs doxorubicin (DOXO) or gentamicin (GENT). **B** Brightfield images showing kOs at 0 h and after 72 h exposure with 2 µM doxorubicin or 1 mg/ml gentamicin. DMSO was used as vehicle control. **C**-**D** Cell viability was evaluated on damaged-kOs using the CellTiter-Glo™ Luminescent Cell Viability Assay (left) and the LDH-Glo™ Cytotoxicity Assay (right) (*n* = 4). **E** Heatmap showing RT-qPCR gene expression of selected genes of interest in kOs treated with 2 µM DOXO and 1 mg/ml GENT. The color scale legend shows minimum and maximum raw values. Data normalized to RPS18 (*n* = 3). **F** Immunofluorescence panel showing whole-mounts of injured-kOs stained for glomerular structures (NPHS1, red), proximal tubules (LTL, green) and the apoptotic marker cleaved CASP3 (white). Nuclei are marked in blue. **G**-**H** Representative immunofluorescence images and pixel quantification of CD31 (green) in damaged kOs (*n* ≥ 6 ROIs of *n* = 2 kOs). **I**-**J** Representative immunofluorescence images and pixel quantification of cleaved CASP3 (white) in damaged kOs (*n* ≥ 7 ROIs of *n* = 3 kOs). **K** Brightfield images of kOs after 72 h drug incubation overlayed with TRITC-albumin images (red). **L** Histograms summarize the machine learning segmentation of albumin signal in pixels (*n* = 12 from 2 independent batches). Results show mean ± SD; *p* < 0.01, ANOVA results of Kruskal-Wallis test followed by multiple comparisons. Scale bars: 200 μm

kOs were generated independently of the cMTs and kept in culture until d7 + 14 to allow structural and functional maturation prior to injury induction (Fig. 1A). We applied two nephrotoxic drugs, 2 µM doxorubicin (DOXO) and 1 mg/ml gentamicin (GENT) and assessed cell viability upon exposure of kOs for 72 h (Fig. 1B-D). Low drug concentrations were selected for co-culture experiments to balance measurable toxicity with partial tissue preservation. Drug concentrations were selected according to previous work in hiPSC-derived kOs [19–22]. The selected dosages resulted in partial impairment of renal tissue structure while eliciting injury signatures such as apoptosis, oxidative stress, inflammation, and early fibrotic remodeling [23]. Progressive morphological deterioration of kOs was evident by brightfield microscope inspection in response to the drugs, with dimethyl sulfoxide (DMSO) serving as the vehicle control (Fig. 1B). To investigate the impact of both DOXO and GENT on treated kOs, we analyzed intracellular ATP using CellTiter-Glo™, which revealed a lower cell viability at 72 h compared to control (Fig. 1C), and quantified release of lactate dehydrogenase in the supernatant using the LDH-Glo™ assay, which indicated increased membrane damage (Fig. 1D).

To further characterize the effects of DOXO and GENT on kOs, we analyzed a selection of kidney-specific and injury-associated genes by RT-qPCR and immunofluorescence analysis (Fig. 1E-F). DOXO led to the downregulation of podocyte markers nephrin (*NPHS1*), podocalyxin (*PODXL*) and synaptopodin (*SYNPO*), while hepatitis A virus cellular receptor 1 (*HAVCR1*, encoding kidney injury molecule 1, KIM-1) was upregulated, indicating structural and epithelial (tubular) damage (Fig. 1E), which was also evident from the reduced LTL signal in DOXO-exposed organoids in Fig. 1F. Reduced CD31 signal indicated also endothelial damage in response to DOXO and GENT treatments (Fig. 1G-H). Exposure to GENT selectively induced upregulation of the oxidative stress marker heme-oxygenase 1 (*HMOX1*). Both nephrotoxic compounds increased the expression of the inflammatory cytokine interleukin 6 (*IL-6*), with DOXO eliciting the strongest response. Lastly, fibrotic markers fibroblast growth factor 23 (*FGF23*) and transforming growth factor beta 1 (*TGFβ1*) were upregulated in damaged kOs in response to both treatments, with a more pronounced induction following DOXO exposure. Although transcript levels of caspase 3 (*CASP3*) were not altered in either treatment condition at 72 h (Fig. 1E), immunofluorescence analysis followed by quantification (Fig. 1F, I, and J) showed a significant increase in cleaved caspase-3 signal in DOXO-treated kOs at d7 + 17, suggesting activation of the apoptotic process at protein level. These findings confirm that both nephrotoxic compounds induce molecular signatures consistent with renal injury, involving cell death, inflammation, oxidative stress, and early fibrotic remodeling.

To assess the functional integrity of kOs following nephrotoxic injury, we investigated the uptake of TRITC-conjugated albumin, a substrate normally transported by kidney proximal tubules [24] (Fig. 1K). Live imaging of whole-mount kOs indicated increased TRITC levels in DOXO and GENT-treated kOs compared to controls, suggesting altered albumin handling in these organoids. This was supported by machine-learning segmentation of the TRITC-albumin signal, revealing higher number of TRITC-positive pixels in damaged kOs, consistent loss of epithelial integrity following injury (Fig. 1L).

### Co-culture of injured kidney organoids with cardiac microtissues leads to cardiac damage

**Fig. 2:**
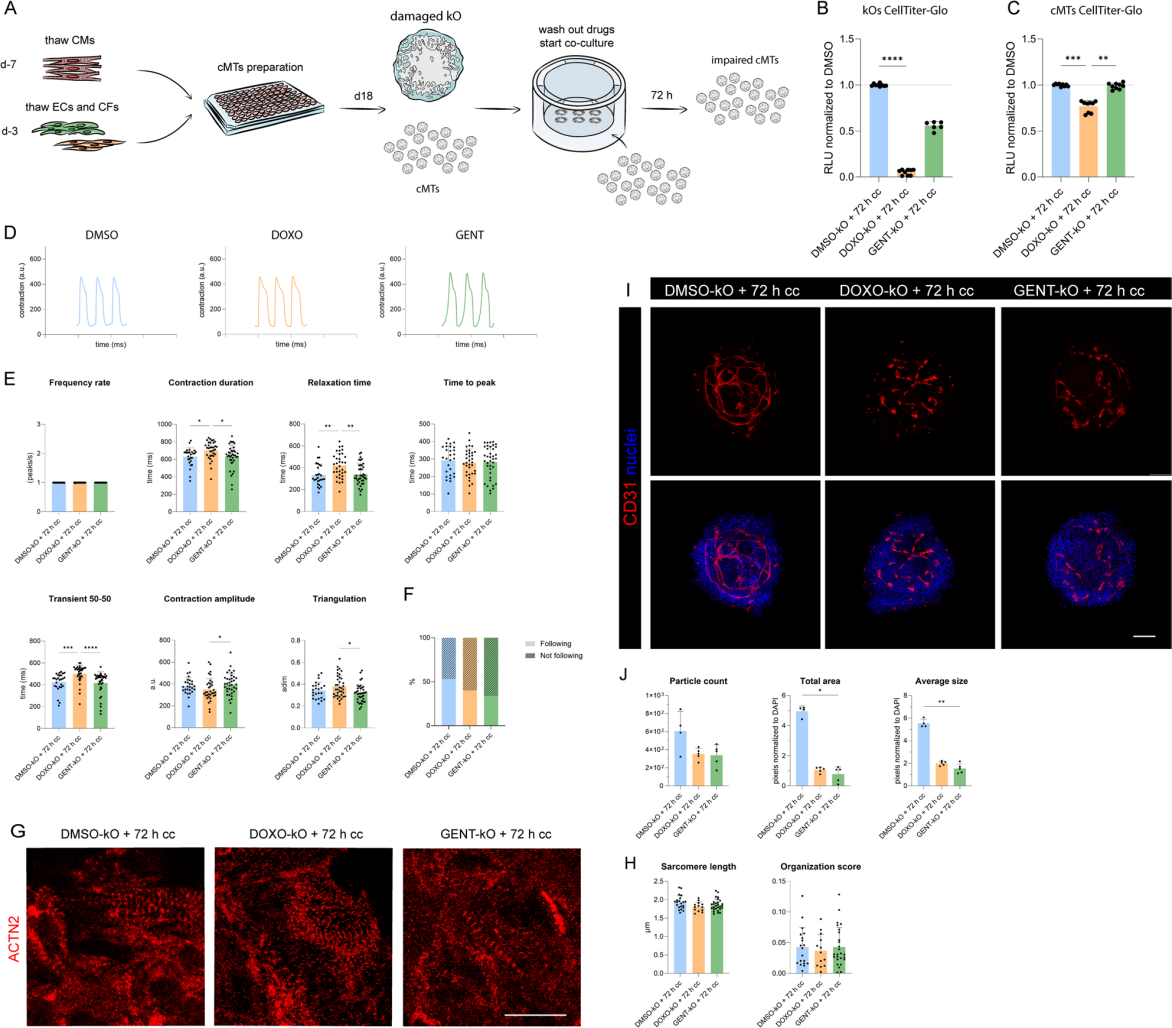
Co-culture of injured kOs leads to indirect cMTs contraction changes and loss of endothelial cells. **A** Schematic representation of the experimental workflow showing cMTs formation by assembling three cardiac cell types (cardiomyocytes or CMs, endothelial cells or ECs, and cardiac fibroblasts or CFs) followed by co-culture with injured kOs in TransWells for 72 h. **B**-**C** Data showing results for cell viability assays using CellTiter-Glo™ on 72 h co-cultured damaged-kOs and cMTs, respectively (*n* ≥ 6). **D** Representative contraction traces for cMTs among indicated conditions. **E** Bar graphs showing quantification of contraction traces parameters such as contraction duration, relaxation time, time to peak, transient 50–50, contraction amplitude and triangulation in cMTs paced at 1 Hz (*n* ≥ 24). **F** Quantification of cMTs following/not following 1 Hz pacing (*n* ≥ 24). **G** Immunofluorescence images showing a close-up of sarcomeres (ACTN2, red) in cMTs after 72 h co-culture with damaged-kOs. Scale bar: 20 μm. **H** Bar graphs showing sarcomere length (left) and organization score (right) (*n* ≥ 13). **I** Immunofluorescence images showing ECs (CD31, red) in cMTs after 72 h co-culture with damaged-kOs. Scale bar: 100 μm. **J** Bar graphs showing quantification of CD31-positive signal (*n* ≥ 4). Nuclei are marked in blue. Results show mean ± SD; *p* < 0.01, ANOVA results of Kruskal-Wallis test followed by multiple comparisons

To investigate the impact of kidney injury on cardiac function, cMTs were generated and matured until d18, then co-cultured for 72 h with either pre-damaged or control kOs (Fig. 2A). A kidney-to-heart cell number ratio of 2:1 was chosen. After inducing damage for 72 h, kOs were washed to remove residual drug and static co-culture was set up by placing cMTs floating in the lower well of the Transwells. After 72 h of co-culture, all tissues were retrieved for further analysis. The viability of injured kOs was lower for both treatments compared to control, indicating the injury on kOs was irreversible (Fig. 2B). In contrast, when compared to control, viability of cMTs was not affected in tissues co-cultured with GENT kOs but was significantly lower for the cMTs in culture with DOXO kOs (Fig. 2C).

The contraction profile of cMTs was then examined after 72 h of co-culture, by pacing them at 1 Hz (Fig. 2D). More cMTs failed to follow pacing at 1 Hz after co-culture with damaged kOs (Fig. 2F), suggesting that damage of the cMTs affected their contractility. Data from cMTs that did respond to pacing, which ensured consistency in conditions that contractile performance was assessed, showed that cMTs co-cultured with DOXO-treated kOs exhibited prolonged contraction duration, delayed relaxation time and increased time to reach 50% of contraction peak (Transient 50–50), indicating slower and impaired contractile response (Fig. 2E). In contrast, time to peak and contraction amplitude remained unaltered between control and treated co-culture conditions. Notably, contraction peak triangulation, a parameter like the action potential triangulation, which indicates pro-arrhythmic propensity when increased, was statistically different only between the DOXO and GENT kO co-cultured cMTs.

To rule out direct drug effects on cMTs we assessed cMT exposed to increasing concentrations of doxorubicin and gentamicin (Figure S1A-B). Fluorescence in co-cultured cMTs was markedly lower than in directly exposed cMTs, indicating minimal drug uptake. Consistently, cMTs directly treated with 2 µM DOXO or 1 mg/ml GENT failed to maintain pacing and triangulation was reduced in both DOXO and GENT treated cMTs (Figure S1C) compared to control. This demonstrates that direct drug exposure impairs contractile performance of cMTs differently compared to co-culture with kOs, confirming that direct and indirect administration of the drugs have distinct effects. These findings suggest that DOXO kOs strongly affect cMTs viability and contraction properties.

To assess whether co-culture with damaged kOs affected sarcomeric organization in CMs within cMTs, we analyzed the α-actinin (ACTN2) signal after 72 h of co-culture. Well-preserved sarcomeric structures (Fig. 2G) with both sarcomeric length and organization score (Fig. 2H) remained unaltered in cMTs co-cultured with damaged-kOs across all conditions. Together, these findings suggest that functional alterations were not caused by changes in the sarcomeric architecture of cardiomyocytes which remains intact upon short-term exposure to damaged kOs.

To investigate whether kidney injury affected cardiac endothelial integrity, whole mount immunofluorescence of cMTs was performed after 72 h of co-culture with DMSO and DOXO/GENT-damaged kOs. This revealed disruption of the endothelial network in cMTs co-cultured with damaged kOs, as suggested by the scattered signal for the EC-specific marker CD31, compared to the well-organized network observed in control co-cultures (Fig. 2I). Quantitative analysis using machine learning segmentation of the CD31 signal showed a reduction in CD31-pixel number in cMTs co-cultured with damaged-kO, indicating a loss of cardiac ECs as indirect consequence of drug-induced injury (Fig. 2J). These findings suggest that injury to kOs can adversely affect the endothelial compartment of cMTs via inter-organ signaling.

### Transcriptional analysis suggests crosstalk between damaged-kidney organoids co-cultured and cardiac microtissues

**Fig. 3:**
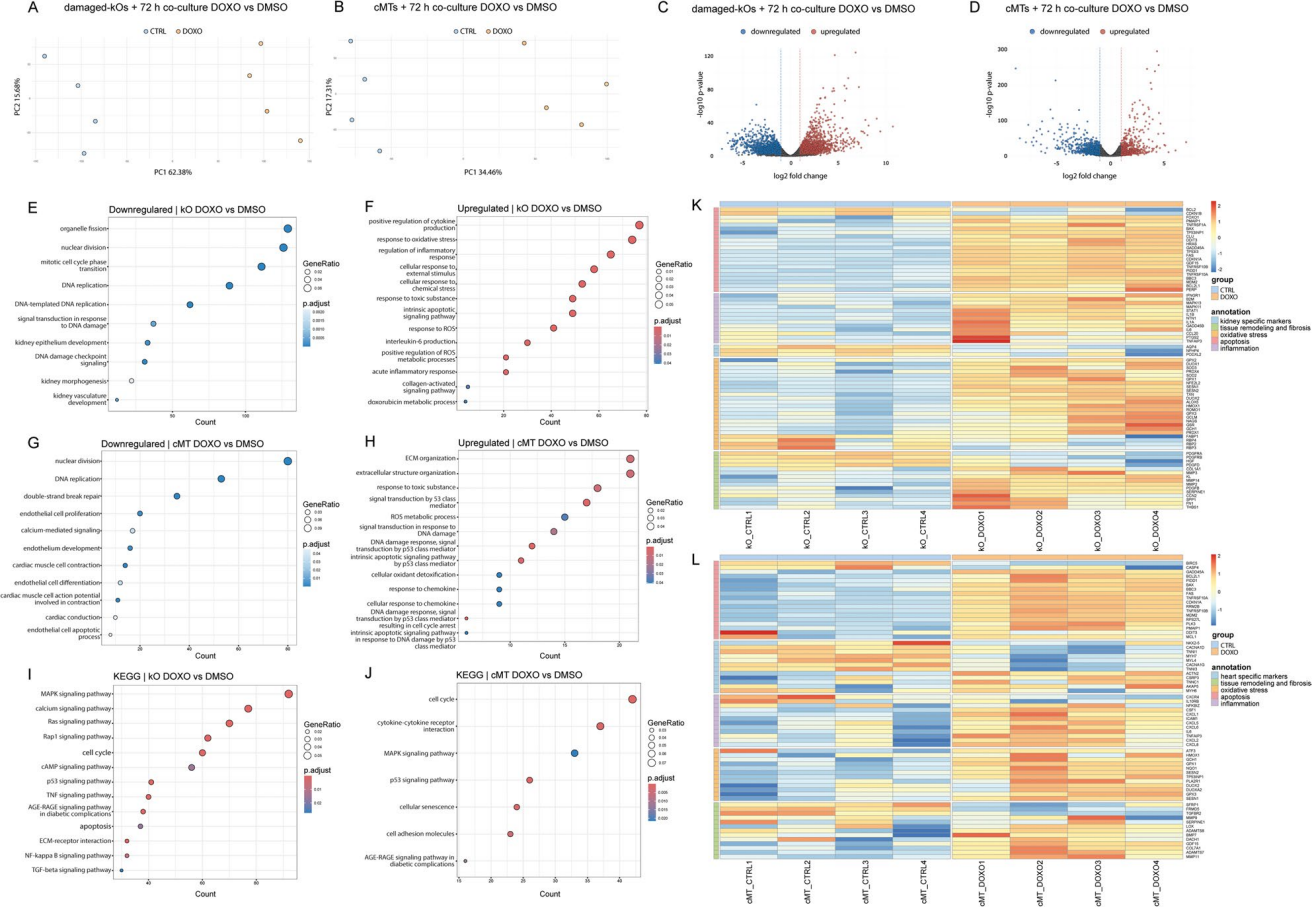
Transcriptomic analysis of doxorubicin-damaged kidney organoids and cardiac microtissues after 72 h co-culture. **A**-**B** PCA analysis of DOXO kOs and co-cultured cMTs compared to DMSO control. **C**-**D** Volcano plots showing downregulated (blue) and upregulated (red) genes in damaged-kOs and cMTs after 72 h co-culture. Genes were considered significantly regulated where log counts per million was less than 0, log fold change greater than 1 or -1 and q-value less than 0.05. **E**-**F** GO biological process terms for significantly downregulated and upregulated genes in co-cultured DOXO kOs. **G**-**H** GO biological process terms for significantly downregulated and upregulated genes in co-cultured cMTs. The p-value and q-value cutoff was set to 0.05 for all GO enrichments and the x-axis represents the number of overlapping genes enriched in each term.** I-J **KEGG pathway analysis for damaged-kOs and cMTs, respectively. **K**-**L** Heatmaps showing altered genes involved in the deregulation of pathways linked to cell apoptosis, inflammation, oxidative stress, tissue remodeling and fibrosis. Specific renal and cardiac markers are also indicated. Samples from *n* = 4 biological replicates per tissue model, per condition. Heatmaps are representative of z-scored log counts per million values.

To profile the effect of the nephrotoxic drugs on the kOs and the impact of their co-culture on cMTs, we performed transcriptome analysis with bulk RNA-sequencing after 72 h co-culture (Supplementary Tables 1–4). Samples from DMSO-treated control, 2 µM DOXO kO and 1 mg/ml GENT kO co-cultures were evaluated for both models. In line with previous results, DOXO showed to have a strong effect on co-cultured tissues, as evidenced from the clustering of the PCA plots for DOXO in both kOs and cMTs (Fig. 3A and B), where treated groups were clearly separated from the control on the first principal component. GENT kOs also clustered separately, whilst cMTs co-cultured with GENT kOs did not separate well based on PCA plots (Figure S2A-B). Heatmaps of the top 100 most variable genes supported these findings, where opposite trends of gene expression could be clearly identified between DOXO kOs and co-cultured cMTs compared with GENT counterparts (Figure S2C-D). A greater response to treatment with DOXO compared to GENT was observed in injured kOs, where 4395 genes were differentially expressed when treated with DOXO and 158 genes when treated with GENT (Fig. 3C and S2E). Notably, most of the genes were downregulated in kOs in response to GENT (Figure S2E). Similarly, 1663 genes were differentially regulated in cMTs co-cultured with DOXO kOs, while only 11 genes were significantly altered when cMTs were co-cultured with GENT kOs (Fig. 3D and S2F). The milder transcriptional response in cMTs compared to kOs was expected, as cMTs were not directly exposed to the drugs; rather, the observed changes likely reflect the indirect effects of injured kOs and possibly the molecular crosstalk between the two organs in co-culture.

We then performed gene ontology (GO) enrichment analysis to classify genes and analyze the altered biological processes in both DOXO kOs and cMTs after co-culture (Supplementary Tables 5–8). The GOs terms of most upregulated and downregulated genes for co-cultured DOXO kOs and cMTs are shown in Fig. 3E-H. As an example, genes associated with cytokine production, oxidative stress, and inflammatory response were upregulated in DOXO kOs upon co-culture, while genes marking mitotic division and DNA replication were mainly downregulated. Moreover, markers of kidney development were also downregulated. For the cMTs co-cultured with DOXO kOs, genes involved in extracellular matrix organization, oxidative stress, DNA damage, and response to chemokine were upregulated, while ECs processes and specific cardiac contraction/conduction and calcium handling genes were downregulated. In general, these genes were involved in the deregulation of pathways belonging to categories linked to cytokine-cytokine receptor interaction, cell apoptosis, inflammation, oxidative stress, tissue remodeling and fibrosis, as displayed in the KEGGs for both models (Fig. 3I-J). Figure 3K-L shows more detail of the most upregulated and downregulated genes belonging to these selected categories for both kOs and cMTs. Specifically, the heatmap in panel K highlights genes modulated in apoptosis, inflammation, oxidative stress, tissue remodeling and fibrosis for kOs, with the addition of kidney specific markers of interests *NPHP4*, *AQP4* and *PODXL2*. Cardiac specific markers shown in panel L include instead *TNNI*, *ACTN2* and *MYH7*. Supplementary Fig. 2G depicts instead the biological processes linked to most downregulated genes for co-cultured GENT kOs, such as cell fate commitment and response to FGF stimulus. Notably, only few genes were upregulated but all of them are associated with renal absorption (*AKR1C3*, *SLC5A2*).

Transcriptomic analysis revealed that DOXO kOs and their paired co-cultured cMTs shared upregulation of multiple pathways associated with apoptosis (*BAX*, *CASP3*,* BCL2L1*), inflammation (*IL6*, *CXCL8*, *CCL2*) and oxidative stress (*HMOX1*, *NQO1*), while fibrosis/tissue remodeling markers (*TGFB1*, *COL1A1*, *MMP2*, *MMP9*) reached statistical significance primarily in the kidney compartment. DOXO kOs revealed robust activation of stress and injury programs, including oxidative stress, cytokine production, IL-6 secretion, acute inflammatory response, apoptosis, collagen-activated signaling, and P53/MAPK/AGE-RAGE pathways, hallmarks of proximal tubular injury. These changes were accompanied by downregulation of proliferative and developmental processes such as nuclear division, DNA replication, and kidney morphogenesis, indicating a shift from growth toward stress-adaptive and inflammatory phenotypes. In co-cultured cMTs, transcriptomic signatures converged on DNA damage signaling via P53, apoptosis, extracellular matrix organization, chemokine and ROS responses, and cell adhesion pathways. Downregulated pathways, in line with our tissue characterization after 72 h co-culture, prominently included cardiac muscle contraction, calcium-mediated signaling, conduction, and endothelial cell proliferation/differentiation. Altogether, transcriptomics analysis confirmed a strong nephrotoxic effect of DOXO on kOs and a consequent cardiac damage because of the co-culture. Of note, upregulation of cytokine production in the injured kOs along with response to chemokine in the co-cultured cMTs suggests a crosstalk between the two co-cultured organoids.

## Discussion

Interorgan communication is a critical determinant of disease progression, yet human experimental systems allowing the study of kidney-to-heart signaling under controlled conditions remain scarce. Here, we leverage state-of-the-art hiPSC-derived kidney and cardiac organoids in a co-culture setting allowing reciprocal crosstalk to capture the cardiac effects caused by drug-induced kidney damage. Using a co-culture system, we show that DOXO caused kOs damage, which in turn impacted on cMT contraction properties and transcriptional profile, suggesting that paracrine signaling contributes to the kOs-cMTs communication axis.

DOXO and GENT are toxic to podocytes and proximal tubules in the kidney and both drugs are taken up by proximal tubule cells via the scavenger receptors megalin and cubilin, which are expressed in our organoid model [25]. The effect exerted by DOXO on kOs was more pronounced than GENT, as evident from the reduced cell viability and more prominent de-regulation of injury and apoptosis markers after 72 h of drug exposure. GENT-induced renal dysfunction in kOs was evident from altered albumin uptake, indicating impaired proximal tubular reabsorption and a “leaky” epithelial barrier [26, 27].

The selection of the experimental design, based on a static Transwell co-culture for kOs and cMTs was chosen for two reasons. Firstly, it allowed controlled exposure of kOs to a dose range of drugs to the renal compartment and characterization of renal injury prior to the setting-up of the co-culture with cMTs and investigating heart-kidney crosstalk. Using this method, we could assess the secondary effects on the cMTs. Secondly, it was possible to retrieve both kOs and cMTs without any tissue damage, enabling assessment of viability, imaging, functional and bulk RNA-seq readouts. A kidney-to-heart ratio of 2:1 (based on cell number) was chosen to approximate physiological conditions. This ensured that the kidney acts as the predominant source of injury mediators during acute renal damage caused by the drugs, while preserving robust cardiac readouts. This design builds on our earlier heart-kidney platform and organ-on-chip (OoC) integration, but goes beyond system integration to interrogate injury-driven interorgan signaling [14].

By co-culturing damaged hiPSC-derived kOs with independently matured hiPSC-derived cMTs, we showed that renal injury alone is sufficient to induce functional and cellular pathology in cardiac tissues. Functionally, the contraction profile of paced cardiac tissues was impaired despite maintaining contraction amplitude. This is consistent with early dysregulated electromechanical signaling rather than sarcomeric disarray [28, 29]. In addition, the selective loss of cardiac ECs aligns with endothelial-first injury pattern where inflammatory cytokines, oxidative stress, and uremic toxins such as indoxyl sulfate and p-cresyl sulfate preferentially damage cardiac endothelium, increase ROS, and impair nitric oxide bioavailability before direct CM injury [30].

DOXO produced a stronger phenotype than GENT in both tissue models, matching the magnitude of transcriptional remodeling in kOs and severity of secondary cardiac changes in co-cultured cMTs. Transcriptomic profiling revealed that DOXO-exposed kOs and their co-cultured cMTs shared activation of apoptosis, inflammatory, and oxidative stress pathways, while fibrosis and tissue remodeling markers were most prominent in the kidney compartment. DOXO kOs displayed robust injury programs, including oxidative stress, cytokine/IL-6 production, apoptosis, and P53/MAPK/AGE-RAGE signaling, consistent with proximal tubular injury hallmarks [31–33]. In contrast, co-cultured cMTs showed convergence on DNA damage signaling, apoptosis, ECM remodeling, chemokine and ROS responses, alongside suppression of cardiac contraction, calcium signaling, and endothelial proliferation, reflecting a shift from physiological function toward stress-adaptive phenotypes. Notably, injured kOs upregulated FGF23, a known driver of cardiac hypertrophy and fibrosis via FGFR4/PLCγ/calcineurin-NFAT signaling [34], while the cMT compartment recapitulated uremic toxin-linked hallmarks, including IL6/CXCL8-driven inflammation, BAX/CASP3 apoptosis, NQO1 induction with NOS3 suppression, and endothelial adhesion remodeling. The absence of strong induction of classic fibrosis genes in cMTs suggests that, at this early phase, the effects caused by the damaged kOs mainly caused endothelial dysfunction and excitation-contraction timing defects rather than structural myocyte failure [35]. Together, these molecular signatures mirror the renal inflammatory/fibrotic stress driving downstream cardiac endothelial dysfunction and contractile slowing.

Consistent with our functional results, GO enrichment of the most upregulated GENT-responsive genes in kOs highlighted renal absorption and transport processes, pathways that reflect the well-established biology of megalin/cubilin-mediated endocytosis and ClC-5-dependent megalin recycling in proximal tubule albumin and aminoglycoside handling. This mechanism is directly exploited by gentamicin to enter cells and disrupt lysosomal integrity. In line with our findings, prior studies have shown that pharmacological or molecular interference with this pathway, for example, blocking megalin with receptor-associated protein (RAP) or soluble RAP (sRAP), markedly reduces GENT accumulation and tubular injury. Similarly, montelukast-mediated downregulation of ClC-5 impairs megalin recycling, decreases aminoglycoside uptake, and ameliorates renal functional and histological damage [26, 27]. Consistent with this more localized and mechanistically targeted injury, the GENT kOs elicited a milder secondary phenotype in co-cultured cMTs, with subtler alterations than those observed with DOXO injury. The cardiac response was characterized by modulated inflammatory signaling and potential activation of stress-adaptive and survival pathways [36–38]. Our study demonstrates that injury confined to the kidney can induce distinct cardiac phenotype through indirect organ signaling, while the specific signaling pathways mediating this kidney-heart communication remain to be elucidated.

Our work establishes a human, stem cell-derived organoid model that captures kidney-to-heart injury propagation, offering a controllable in vitro platform that may recapitulate key features of CRS type 3, where acute kidney injury precipitates secondary cardiac dysfunction [1, 2]. Clinically, CRS type 3 often manifests with early cardiac endothelial dysfunction driven by systemic inflammation, oxidative stress and exposure to uremic toxins, which lead to impaired nitric oxide signaling and microvascular injury. These changes precede myofibrillar damage, but impair excitation-contraction coupling and relaxation dynamics in the heart [39]. The separation between endothelial vulnerability and myofibrillar integrity argues that early CRS type 3 signaling preferentially targets the vascular compartment, while CM structure remains initially preserved, consistent with progression from endothelial dysfunction to contractile impairment [28]. Transcriptional suppression of contractile and vascular maintenance programs aligns with our functional findings of impaired contraction kinetics and endothelial loss, and mirrors early CRS type 3 pathophysiology in which inflammatory and oxidative cues from the injured kidney precede myocyte structural failure [27–29].

While this study establishes a model for investigating early kidney-to-heart injury, with the current 72 h observation window capturing an early sub-acute phase of CRS-like signaling, some limitations should be acknowledged: (i) shorter exposure pulses may help resolve the dynamics of acute mediator release [40], whereas extended co-cultures could reveal chronic remodeling and progressive myocyte structural decline, as observed in the cardiac transcriptome of in vivo CRS type 4 models [6]; (ii) the absence of hemodynamic forces, vascular shear, and clearance mechanisms in our static configuration; this could be introduced using OoC microphysiological systems, which have been shown to sustain functional coupling between multiple human tissues and capture physiologically relevant interorgan crosstalk [41, 42]. Physiological flow is known to modulate endothelial activation, inflammatory signaling, and toxin clearance, and may therefore quantitatively influence the magnitude and kinetics of injury propagation observed in this study [43]; (iii) while our data highlight plausible mediators of injury transmission, establishing causality would require systematic secretome profiling of cytokines, chemokines, and uremic toxins to prove paracrine signalling; although cytokine-mediated signaling is shown, the observed responses are likely driven by multiple inflammatory and stress-related mediators rather than a single dominant cytokine, consistent with the complex paracrine environment described in kidney-heart injury. Additionally, targeted loss-of-function experiments such as FGF23/FGFR blockade, ROS scavenging, and megalin-pathway modulation would further dissect the molecular mechanisms [26, 44, 45]; (iv) expansion to multiple donor lines and patient-derived hiPSCs may enable interrogation of genetic susceptibility and pharmacologic response heterogeneity, increasing the translational relevance of the platform.

By combining two hiPSC-derived organoids in a modular and scalable format, this platform provides a versatile tool for both basic and translational research. Its ability to reproduce kidney-to-heart injury transmission creates opportunities for drug screening as well as for mechanistic studies of interorgan signaling pathways. Moreover, the system may be suited for preclinical testing of candidate therapeutics aimed at halting injury propagation, such as anti-inflammatory or anti-fibrotic biologics, endocytosis inhibitors, and endothelial-protective agents. In a landscape where controllable human models of CRS type 3 are virtually absent, our model fills a critical experimental gap and offers a flexible foundation that could be readily adapted to flow-based microphysiological platforms to further enhance physiological relevance and clinical translatability.

## Conclusion

Our findings show that kidney injury in hiPSC-derived organoids can drive early functional and structural changes in co-cultured cardiac microtissues, including endothelial loss and impaired contractile dynamics, while cardiomyocyte architecture remains initially preserved. By combining controlled induction of renal damage with a human heart-kidney co-culture system, we reproduce key features of kidney-to-heart injury transmission observed in CRS type 3. The modular and scalable nature of this platform enables the study of interorgan signaling pathways and provides opportunities for screening therapeutic strategies aimed at preventing or reversing secondary cardiac damage following AKI.

## Materials and methods

### Cell lines

We used hiPSC lines previously generated and approved by the medical ethical committee at the Leiden University Medical Center: LUMC0020iCTRL-06 (https://hpscreg.eu/cell-line/LUMCi028-A [46–48]), and LUMC_GMP_hiPSC D2 L1-B [49].

### Cell differentiation and generation of cardiac microtissues

cMTs were generated as previously from hiPSC-derived cardiomyocytes (CMs), cardiac fibroblasts (CFs) and cardiac endothelial cells (ECs) [48]. Differentiated and cryopreserved cardiac cells were thawed 3–5 days before cMT formation and a total of 5 × 10^3^ cells/cMT were combined using a defined ratio of 70:15:15 (CMs: CFs: ECs) in 50 µl in LI-BPEL medium supplemented with 50 ng/ml VEGF (Miltenyi Biotec, Cat. No: 130-109-386) and 5 ng/ml FGF-2 (Miltenyi Biotec, Cat. No: 130-093-842) in V-shaped culture microplates (Greiner Bio-one, Cat. No: 651161). To enhance the aggregation of cells at the bottom of the plates, microplates were centrifuged at 300 $\:\overrightarrow{g}$ for 10 min at room temperature (RT). At day 0 of cMT formation, plates were gently placed in an incubator at 37 °C, 5% CO_2_. Plates were not moved for 3 days to allow proper aggregation of the cells. cMTs were refreshed every 3 days with a partial medium change of 25 µl LI-BPEL supplemented with 50 ng/ml VEGF and 5 ng/ml FGF-2 from day 3 to day 18.

### Cell differentiation and generation of kidney organoids

kOs were generated using hiPSCs as previously described from the adaptation of the protocol by Takasato et al. [25, 50]. In short, 1.5 × 10^5^ cells/cm hiPSCs were plated on vitronectin-coated culture dishes in E8 medium (Thermo Fisher Scientific, Cat. No: A1517001) supplemented with RevitaCell (Thermo Fisher Scientific, Cat. No: A2644501). After 24 h, when cell confluency had reached 10%-20%, differentiation was initiated. Cells were cultured in 6 µM CHIR99021 (R&D Systems, Cat. No: 4423) in TeSR-E6 Basal Medium (E6, Stemcell Technologies, Cat. No: 05947) supplemented with Antibiotic Antimycotic Solution (110X, Sigma Aldrich, Cat. No: A5955) for 4 days. Culture medium was then replaced by E6 medium containing 200 ng/ml rhFGF9 (R&D Systems, Cat. No: 273-F9) and 1 mg/ml heparin (Sigma-Aldrich, Cat. No: 9041-08-1). On day 7, cells were incubated with 5 µM CHIR99021 in E6 for a 1 h pulse and switched from monolayer to 3D culture on Transwell 0.4 μm pore polyester membranes in the same medium. A paste of 200 × 10^3^ cells per µl was prepared and 1 µl droplets were deposited on top of the membrane using an automatic dispenser. On day 7 + 5, growth factors were removed and a 1.2 ml E6 medium change was performed every 2 days. Organoids were cultured at 37 °C, 5% CO_2_ until day 7 + 14.

### Drug-induced damage of kidney organoids

To induce damage, kOs were incubated at day 7 + 14 using E6 medium supplemented with 2 µM doxorubicin (Selleckchem, Cat. No: S1208-10 mg) or 1 mg/ml gentamicin (Centrafarm, Cat. No: RVG 57572) for 72 h. DMSO (Sigma, Cat. No: D2650) was used as vehicle control. After 72 h, kOs on Transwell membranes were transferred to a new 6-wells plate in drug-free E6 medium to start the co-culture experiments.

### Co-culture of cardiac microtissues and kidney organoids

For the co-culture experiments, cMTs were collected from V-shaped culture plates and gently resuspended in LI-BPEL medium. Medium with the tissues was then pipetted in the bottom compartment of the 6 well plate below the Transwell membranes containing kOs, leaving cMTs suspended in medium. Co-culture conditions were kept for 72 h.

### Viability assays

Assays were performed using the CellTiter-Glo™ Luminescent Cell Viability Assay (Promega, Cat. No: G7570). Two kOs and 10 cMTs for each condition were resuspended in a 1:1 ratio of LI-BPEL medium and reagent, respectively. After 10 min of incubation, while protected from light, tissues were thoroughly lysed and triplicates were pipetted onto a flat bottom, opaque white 96-wells plate (Costar, Cat. No: 3912). The LDH-Glo™ Cytotoxicity Assay (Promega, Cat. No: J2380) was also performed, where LI-BPEL culture supernatant and the kit’s reagent were mixed in a 1:1 ratio and incubated for 10 min in the dark. Luminescence for both assays was measured through the SoftMaxPro software on the SpectraMax iD3x microplate reader (Molecular Devices).

### Immunostaining

#### Immunostaining of cardiac microtissues

Immunostaining of cMTs was performed as previously described [48]. Tissues were fixed using 4% paraformaldehyde (PFA, Merck, Cat. No: 104005) for 1 h at 4º C followed by permeabilization in 0.2% Triton X-100 (Sigma-Aldrich, Cat. No: T8787) in PBS^+/+^ (Thermo Fisher Scientific, Cat. No: 14040-091) for 30 min at RT. After washing with PBS^+/+^, samples were blocked with 10% fetal bovine serum (FBS, Sigma-Aldrich, Cat. No: T8787) in PBS^+/+^ for 2 h at RT. Primary antibodies against ACTN2 (mouse monoclonal, Sigma-Aldrich Cat. No: A7811 dilution 1:1000) and CD31 (sheep polyclonal, R&D Systems, Cat. No: AF806, 1:200) were diluted in blocking solution and incubated overnight at 4º C. The following day, tissues were washed 3 times in PBS^+/+^, then incubated for 2 h at RT in blocking solution containing Alexa-Fluor 594 and 488 conjugated antibodies (Thermo Fisher Scientific, Cat. No: A-21203; Thermo Fisher Scientific, Cat. No: A-21206). Nuclei were counterstained with Hoechst (Invitrogen, Cat. No: H3570, dilution 1:10,000). Finally, cMTs were mounted using ProLong Gold Antifade Mountant (Thermo Fisher Scientific, Cat. No: P36930) and imaged using a Dragonfly 200 Series high-speed confocal imaging platform (Oxford Instruments).

#### Immunostaining of kidney organoids

kOs were fixed and stained as previously described [25, 50]. Briefly, tissues were fixed in 4% PFA for 30 min at 4º C. Permeabilization and blocking of kOs were performed in a solution of 0.3% Triton X-100 and 10% donkey serum in PBS^+/+^ for 2 h at RT. kOs were then incubated overnight at 4º C with primary antibodies against NPHS1 (sheep polyclonal, R&D Systems, Cat. No: AF4269, dilution 1:100), LTL (biotinylated, Vector Laboratories, Cat. No: B-1325-2, dilution 1:300), CD31 (mouse, DAKO, Cat. No: M0823, 1:100) and CASP3 (rabbit, Cell Signaling, Cat. No: 9661 S, 1:100) in blocking solution. The following day, tissues were washed 3 times in 0.3% Triton X-100 in PBS^+/+^, then incubated for 2 h at RT in blocking solution containing Alexa-Fluor 488, 568, 594 and 647 secondary antibodies (Thermo Fisher Scientific, Cat. No: A21202, A21099, A11005, A21448) and streptavidin–Alexa Fluor 488 (Thermo Fisher Scientific, Cat. No: S11223). Nuclear staining was performed with Hoechst 33,342 (Thermo Fisher Scientific, Cat. No: H3570, dilution 1:10,000). Finally, kOs were mounted onto 35 mm glass bottom dishes (MatTek, 14 mm microwell No. 1.5 coverglass, Cat. No: P35G-1.5-14-C) with glycerol or ProLong Gold Antifade Mountant. Imaging was performed as described above or using the Leica White Light Laser Confocal Microscope TCS SP8 coupled to LAS-X imaging software.

### MUSCLEMOTION analysis of cardiac microtissues

Contraction video analysis was performed using the MUSCLEMOTION software, as previously described [51]. Briefly, videos of cMTs were recorded for 10 s at 100 frames per second (fps) using a 10x objective (Nikon Eclipse Ti inverted microscope, ThorLabs DCC3240M camera) under controlled conditions (37° C, 5% CO_2_). Tissues were electrically paced at 1 Hz (20 V, 3 ms pulse width) using custom-made electrodes designed to fit V-shaped wells [48]. cMTs that failed to respond to applied pacing condition were excluded from the analysis. Quantified parameters of contraction traces included contraction duration, time to peak, relaxation time, 50-to-50 transient, and contraction amplitude. The contraction triangulation (analogous to action potential triangulation) was calculated as the ratio of the 10-to-10 transient to the 90-to-90 transient, prior to averaging. Contraction frequency was determined by dividing 1000 ms by the peak-to-peak interval.

### Quantification of sarcomeric length and organization

Quantification of sarcomeric length and organization score of cMTs after 72 h of co-culture with damaged kOs was performed using the SotaTool [52]. Fixed cMTs were stained for ACTN2, mounted and imaged as previously described. Regions of interest (ROIs) from one plane of each cMTs per condition were analyzed.

### Live imaging of TRITC-conjugated albumin uptake in kidney organoids

For albumin uptake assays, kOs were incubated overnight at 37° C and 5% CO_2_ in 300 µl E6 containing 10 µg/ml TRITC-conjugated albumin (10 mg/ml stock, Sigma Aldrich, Cat. No: A2289) [53]. Following incubation, organoids were washed 3 times with PBS^+/+^ and transferred in glycerol to 35 mm MatTek glass-bottom dishes for live imaging. Imaging was performed using an SP8WLL Inverted fluorescent confocal laser-scanning microscope (Leica Microsystems, Model No: TCS SP8 X equipped with White Light Laser).

### Segmentation analysis using Labkit

Machine learning segmentation was performed using the Fiji plug-in Labkit. Separate classifiers were trained for the detection of TRITC-albumin, CASP3 and CD31 signals. Each classifier was applied to its respective fluorescence channel to distinguish positive signal from background. A custom macro was developed and used to enable image processing and batch analysis across multiple samples.

### RNA extraction

The RNeasy Micro Kit (Qiagen, Cat. No: 74004) and the NucleoSpin^®^ RNA/Protein kit (Macherey-Nagel, Cat. No: 740933.250) were used for RNA extraction of cMTs and kOs, respectively. Samples were quantified using the NanoDrop One Spectrophotometer (Thermo Fisher Scientific).

### Quantitative reverse transcription PCR (RT-qPCR)

cDNA was synthesized from 250 ng total RNA using the iScript cDNA Synthesis Kit (Bio-Rad, Cat. No: 1708890) and Bio-Rad S1000 Thermal Cycler. Primers for genes of interest were designed and ordered through the Integrated DNA Technology platform (primer sequences are listed in Table 1). Quantitative PCR (qPCR) reactions were prepared using iQ SYBR Green Supermix (Bio-Rad, Cat. No: 1708880) and run on the Bio-Rad CFX96 Touch Real-Time PCR Detection System. Analysis was performed using the CFX Manager™ software (Bio-Rad). Heatmap was build using GraphPad Prism 8.2.0.

**Table 1:** qPCR oligonucleotide primer sequences.

Oligonucleotides		
Primer sequences for qPCR		Encoded protein
*NPHS1*_FW	AGTGTGGCTAAGGGATTACCC	Nephrin
*NPHS1*_RV	TCACCGTGAATGTTCTGTTCC	Nephrin
*PODXL*_FW	AAGGCCAGGGGTTCACAT	Podocalyxin
*PODXL*_RV	AGCCTCGCATCCCTCTAACT	Podocalyxin
*SYNPO*_FW	GCCGCAAATCCATGTTTACT	Synaptodin
*SYNPO*_RV	CTCATCCGCTGTCTGTACCA	Synaptodin
*HAVCR1*_FW	CTTCACCTCAGCCAGCAGAAAC	Kidney injury molecule 1
*HAVCR1*_RV	GCCATCTGAAGACTCTGTCACG	Kidney injury molecule 1
*CASP3*_FW	CATGGAAGCGAATCAATGGACT	Caspase 3
*CASP3*_RV	CTGTACCAGACCGAGATGTCA	Caspase 3
*HMOX1*_FW	AAGACTGCGTTCCTGCTCAAC	Heme oxygenase 1
*HMOX1*_RV	AAAGCCCTACAGCAACTGTCG	Heme oxygenase 1
*IL10*_FW	TCACATGCGCCTTGATGTCTG	Interleukin 10
*IL10*_RV	GACTTTAAGGGTTACCTGGGTTG	Interleukin 10
*IL6*_FW	ACTCACCTCTTCAGAACGAATTG	Interleukin 6
*IL6*_RV	CCATCTTTGGAAGGTTCAGGTTG	Interleukin 6
*FGF23*_FW	CAGAGCCTATCCCAATGCCTC	Fibroblast growth factor 23
*FGF23*_RV	GGCACTGTAGATGGTCTGATGG	Fibroblast growth factor 23
*TGFβ1*_FW	GGCCAGATCCTGTCCAAGC	Transforming growth beta factor 1
*TGFβ1*_RW	GTGGGTTTCCACCATTAGCAC	Transforming growth beta factor 1

### Bulk RNA sequencing and analysis

Whole-genome transcriptome data were generated at Novogene (Novogene GmbH, Germany) using the NovaSeq X Plus Series (40 M reads). RNAseq reads belonging to kidney kOs or cMTs were processed using the opensource BIOWDL RNAseq pipeline v5.0.0 developed at the LUMC [54]. This pipeline performs FASTQ preprocessing (including quality control, quality trimming, and adapter clipping), alignment, read quantification, and optionally transcript assembly. FastQC (v0.11.9) was used for checking raw read QC. Adapter clipping was performed using Cutadapt (v2.10) with the default settings [55]. RNAseq reads’ alignment was performed using STAR (v2.7.5a) on human reference genome GRCh38 [56]. The gene read quantification was performed using HTSeq-count (v0.12.4) with the stranded setting set to reverse [57]. Raw read counts were further processed using edgeR as suggested by the authors [58]. Briefly, counts were combined with sample metadata containing experimental groups and replicate information followed by creation of an edgeR DGElist object. Genes with counts per million values less than 10 were removed and counts were normalized to the library size differences using the Trimmed mean of the M-values method. The model formula included the differences between groups, namely treatment or control, as well as correction for batch effects in both the kO and cMT reads. This formula was subsequently used to create the design matrix subsequently used to estimate dispersion. The processed RNAseq data was subjected to statistical testing using a generalized linear model likelihood ratio test. The resulting p-values were corrected using the Benjamini-Hochberg method to control the false discovery rate (FDR). Genes were considered significantly regulated if the FDR or p-value was less than 0.05, the log(fold change) greater than − 1 or 1 and a logCPM value greater than 0. Gene lists were further subjected to gene ontology (GO) enrichment and KEGG pathway enrichment analysis using clusterProfiler [59].

### Statistical analysis

Statistical analyses were conducted using GraphPad Prism 8.2.0 and RStudio. For contraction analysis, normal distribution was checked using the D’Agostino & Pearson test and the Kruskal-Wallis one-way analysis of variance followed by multiple comparisons was applied. A p-value < 0.05 was considered statistically significant. Data are presented as the Mean ± SD, with sample sizes specified in the corresponding figure legends.

## Supplementary Information

Supplementary Material 1. Supplementary figure 1. Direct effect of drugs on cMTs after 72 h exposure. (A-B) Brightfield images showing the uptake of DOXO (0.03-2 µM) and GENT (0.1-1 mg/ml) in cMTs damaged for 72 h compared to tissues co-cultured with a damaged kOs. Scale bar: 100 µm. (C) Quantification of cMTs damaged for 72 h with 2 µM DOXO or 1 mg/ml GENT following/not following 1 Hz pacing and triangulation output from MUSCLEMOTION among conditions (n>5).

Supplementary Material 2. Supplementary figure 2. Transcriptional effect of 1 mg/ml gentamicin on co-cultured damaged-kOs and cMTs. (A-B) PCA analysis of GENT kOs and co-cultured cMTs compared to DMSO control. (C-D) Heatmaps of the 100 most upregulated/downregulated genes among conditions for co-cultured damaged-kOs and cMTs, respectively. Heatmaps were generated using a z-scored log counts per million value. (E-F) Volcano plots showing downregulated (blue) and upregulated (red) genes in damaged-kOs and cMTs after 72 h co-culture. Genes were considered differentially regulated where the log counts per million was less than 0, the log fold change was greater than 1 or less than -1 and the q-value was less than 0.05. (G) GO biological process terms for significantly downregulated co-cultured damaged-kOs. Terms were considered enriched where the p- and q-value were less than 0.05 and the x-axis depicts the number of genes enriched in each term. Samples from n=4 biological replicates per tissue model, per condition.

Supplementary Material 3. Supplementary Table 1

Supplementary Material 4. Supplementary Table 2

Supplementary Material 5. Supplementary Table 3

Supplementary Material 6. Supplementary Table 4

Supplementary Material 7. Supplementary Table 5

Supplementary Material 8. Supplementary Table 6

Supplementary Material 9. Supplementary Table 7

Supplementary Material 10. Supplementary Table 8

## Data Availability

The datasets supporting the conclusions of this article are included within the article (and its additional files).
