# Supplementary figures and images for: In vitro modeling of renal injury-induced cardiac effects using human iPSC-derived organoids
Source: Cell Commun Signal. 2026 May 6;24:380. doi: 10.1186/s12964-026-02902-3 (PMC13317422; doi:10.1186/s12964-026-02902-3)

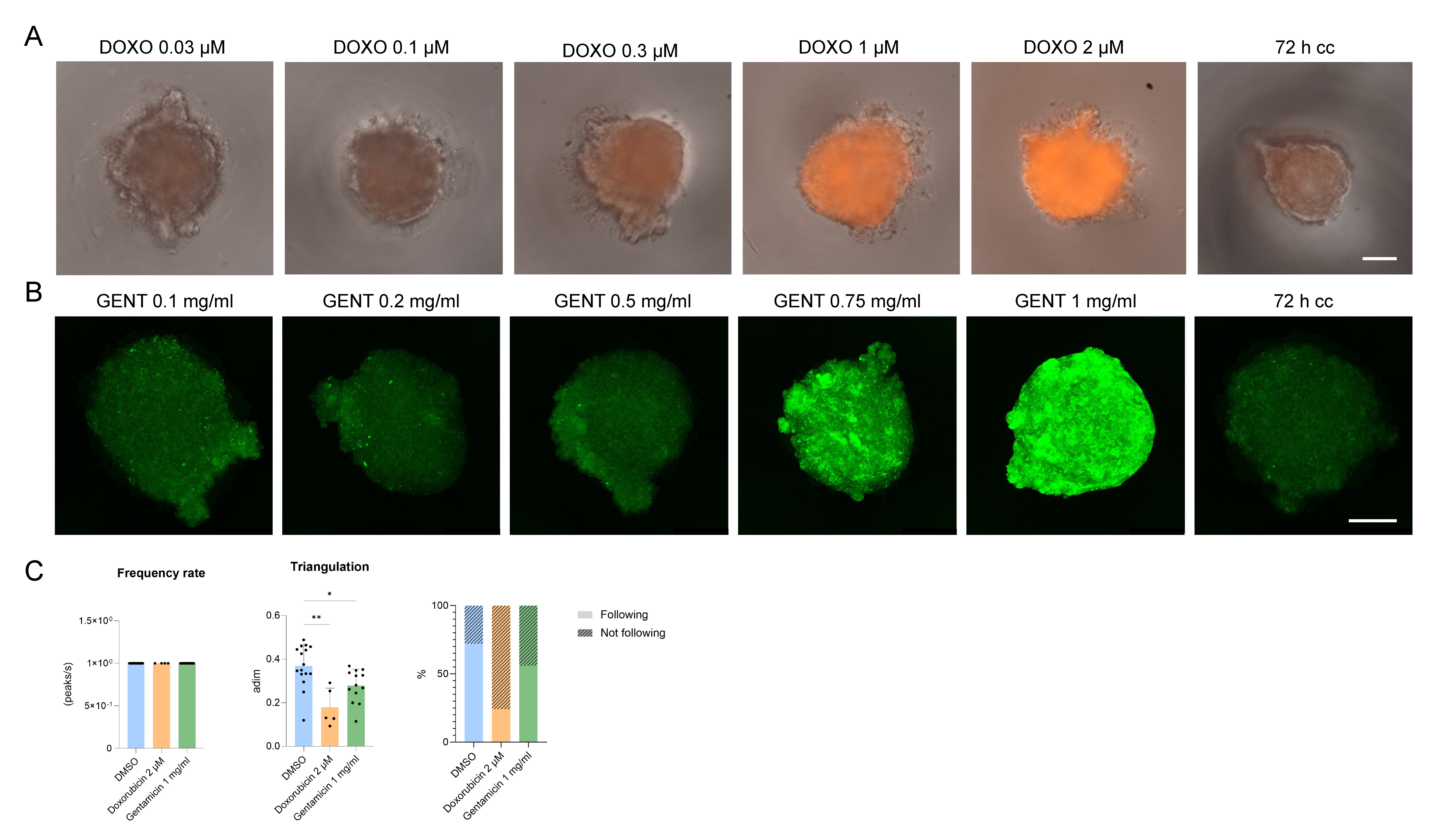

Supplement: Supplementary file 1 — Supplementary Material 1. Supplementary figure 1. Direct effect of drugs on cMTs after 72 h exposure. (A-B) Brightfield images showing the uptake of DOXO (0.03-2 µM) and GENT (0.1-1 mg/ml) in cMTs damaged for 72 h compared to tissues co-cultured with a damaged kOs. Scale bar: 100 µm. (C) Quantification of cMTs damaged for 72 h with 2 µM DOXO or 1 mg/ml GENT following/not following 1 Hz pacing and triangulation output from MUSCLEMOTION among conditions (n>5). [file 12964_2026_2902_MOESM1_ESM.tif]
